# Publisher Correction: Phonon promoted charge density wave in topological kagome metal ScV_6_Sn_6_

**DOI:** 10.1038/s41467-024-46562-8

**Published:** 2024-03-11

**Authors:** Yong Hu, Junzhang Ma, Yinxiang Li, Yuxiao Jiang, Dariusz Jakub Gawryluk, Tianchen Hu, Jérémie Teyssier, Volodymyr Multian, Zhouyi Yin, Shuxiang Xu, Soohyeon Shin, Igor Plokhikh, Xinloong Han, Nicholas C. Plumb, Yang Liu, Jia-Xin Yin, Zurab Guguchia, Yue Zhao, Andreas P. Schnyder, Xianxin Wu, Ekaterina Pomjakushina, M. Zahid Hasan, Nanlin Wang, Ming Shi

**Affiliations:** 1 Photon Science Division, Paul Scherrer Institut, CH-5232 Villigen PSI, Switzerland; 2 Center of Quantum Materials and Devices and Department of Applied Physics, Chongqing University, 401331 Chongqing, China; 3 Department of Physics, City University of Hong Kong, Kowloon, Hong Kong China; 4 City University of Hong Kong Shenzhen Research Institute, Shenzhen, China; 5 Hong Kong Institute for Advanced Study, City University of Hong Kong, Kowloon, Hong Kong China; 6 College of Science, University of Shanghai for Science and Technology, 200093 Shanghai, China; 7 Laboratory for Topological Quantum Matter and Advanced Spectroscopy (B7), Department of Physics, Princeton University, Princeton, NJ USA; 8 Laboratory for Multiscale Materials Experiments, Paul Scherrer Institut, CH-5232 Villigen PSI, Switzerland; 9 International Center for Quantum Materials, School of Physics, Peking University, 100871 Beijing, China; 10 Department of Quantum Matter Physics, University of Geneva, 24 Quai Ernest-Ansermet, 1211 Geneva 4, Switzerland; 11 Advanced Materials Nonlinear Optical Diagnostics lab, Institute of Physics, NAS of Ukraine, 46 Nauky pr., 03028 Kyiv, Ukraine; 12 Institute for Quantum Science and Engineering and Department of Physics, Southern University of Science and Technology of China, Shenzhen, Guangdong 518055 China; 13 Kavli Institute for Theoretical Sciences, University of Chinese Academy of Sciences, 100190 Beijing, China; 14 Center for Correlated Matter and Department of Physics, Zhejiang University, 310058 Hangzhou, China; 15 Department of physics, Southern University of Science and Technology, 518055 Shenzhen, Guangdong China; 16 Laboratory for Muon Spin Spectroscopy, Paul Scherrer Institute, CH-5232 Villigen PSI, Switzerland; 17 Max-Planck-Institut für Festkörperforschung, Heisenbergstrasse 1, D-70569 Stuttgart, Germany; 18 CAS Key Laboratory of Theoretical Physics, Institute of Theoretical Physics, Chinese Academy of Sciences, Beijing, 100190 China; 19 Beijing Academy of Quantum Information Sciences, Beijing, 100913 China; 20 Collaborative Innovation Center of Quantum Matter, Beijing, 100871 China

**Keywords:** Electronic properties and materials, Phase transitions and critical phenomena

Correction to: *Nature Communications* 10.1038/s41467-024-45859-y, published online 23 February 2024

In this article one of the affiliation details for Ming Shi was incorrectly given as ‘Kavli Institute for Theoretical Sciences, University of Chinese Academy of Sciences, 100190 Beijing, China’ but should have been ‘Center for Correlated Matter and Department of Physics, Zhejiang University, 310058 Hangzhou, China’. The original article has been corrected.

