# Supplementary material for: Phonon promoted charge density wave in topological kagome metal ScV6Sn6
Source: Nat Commun. 2024 Feb 23;15:1658. doi: 10.1038/s41467-024-45859-y (PMC10891150; doi:10.1038/s41467-024-45859-y)
Supplement: Supplementary file 1 — Supplementary Information [file 41467_2024_45859_MOESM1_ESM.pdf]

Supplementary Information

Phonon promoted charge density wave in topological kagome metal ScV_6Sn_6

Yong Hu[1,2,#,*], Junzhang Ma[3,4,5,#], Yinxiang Li[6,#], Yuxiao Jiang[7], Dariusz Jakub Gawryluk[8], Tianchen Hu[9], Jérémie Teyssier[10], Volodymyr Multian[10,11], Zhouyi Yin[12], Shuxiang Xu[9], Soohyeon Shin[8], Igor Plokhikh[8], Xinloong Han[13], Nicholas C. Plumb[1], Yang Liu[14], Jia-Xin Yin[15], Zurab Guguchia[16], Yue Zhao[12], Andreas P. Schnyder[17], Xianxin Wu[18,*], Ekaterina Pomjakushina[8], M. Zahid Hasan[7], Nanlin Wang[9,19,20], and Ming Shi[14,1,*]

[1]*Photon Science Division, Paul Scherrer Institut, CH-5232 Villigen PSI, Switzerland*

[2]*Center of Quantum Materials and Devices and Department of Applied Physics, Chongqing University, Chongqing 401331, China*

[3]*Department of Physics, City University of Hong Kong, Kowloon, Hong Kong, China*

[4]*City University of Hong Kong Shenzhen Research Institute, Shenzhen, China*

[5]*Hong Kong Institute for Advanced Study, City University of Hong Kong, Kowloon, Hong Kong, China*

[6]*College of Science, University of Shanghai for Science and Technology, Shanghai 200093, China*

[7]*Laboratory for Topological Quantum Matter and Advanced Spectroscopy (B7), Department of Physics, Princeton University, Princeton, NJ, USA*

[8]*Laboratory for Multiscale Materials Experiments, Paul Scherrer Institut, CH-5232 Villigen PSI, Switzerland*

[9]*International Center for Quantum Materials, School of Physics, Peking University, Beijing 100871, China*

[10]*Department of Quantum Matter Physics, University of Geneva, 24 Quai Ernest-Ansermet, 1211 Geneva 4, Switzerland*

[11]*Advanced Materials Nonlinear Optical Diagnostics lab, Institute of Physics, NAS of Ukraine, 46 Nauky pr., 03028 Kyiv, Ukraine*

[12]*Institute for Quantum Science and Engineering and Department of Physics, Southern University of Science and Technology of China, Shenzhen, Guangdong 518055, China*

[13]*Kavli Institute for Theoretical Sciences, University of Chinese Academy of Sciences, Beijing 100190, China*

[14]*Center for Correlated Matter and Department of Physics, Zhejiang University, Hangzhou 310058, China*

[15]*Department of physics, Southern University of Science and Technology, Shenzhen, Guangdong 518055, China*

[16]*Laboratory for Muon Spin Spectroscopy, Paul Scherrer Institute, CH-5232 Villigen PSI, Switzerland*

[17]*Max-Planck-Institut für Festkörperforschung, Heisenbergstrasse 1, D-70569 Stuttgart, Germany*

[18]*CAS Key Laboratory of Theoretical Physics, Institute of Theoretical Physics, Chinese Academy of Sciences, Beijing 100190, China*

[19]*Beijing Academy of Quantum Information Sciences, Beijing 100913, China*

[20]*Collaborative Innovation Center of Quantum Matter, Beijing 100871, China*

[#]*These authors contributed equally to this work.*

**To whom correspondence should be addressed:*

Y.H. (yonghphysics@gmail.com); X.W. (xxwu@itp.ac.cn); M.S. (ming.shi@psi.ch)

Contents

1. Determination of surface terminations of ScV_6Sn_6

2. K_z dispersion in ScV_6Sn_6

3. $\mathbb{Z}_2$ topological surfaces states in ScV_6Sn_6

4. VHS around the *K* point

5. Orbital character resolved band structure of ScV_6Sn_6

6. Evolution of the Raman modes in ScV_6Sn_6 across the CDW transition

Supplementary Note 1. Determination of surface terminations of ScV_6Sn_6

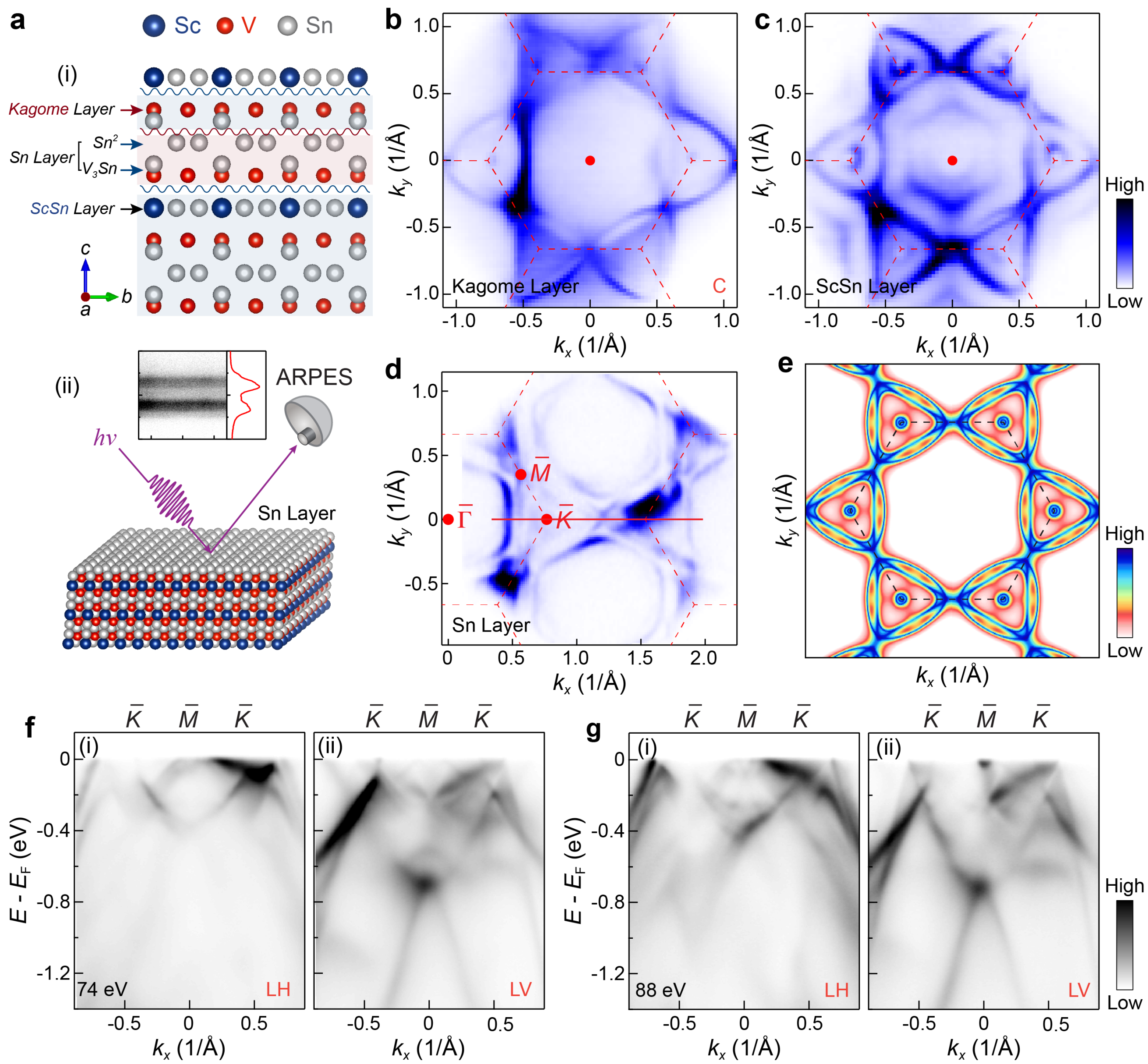

Fig. S1| Electronic structure of the Sn^2 termination. a Crystal structure (side view) of ScV_6Sn_6 showing three possible surface terminations as indicated the arrows (i) and schematic of the Sn termination (ii). **b-d** Fermi surface (FS) mappings collected on the three terminations, measured with circular (C) polarized light. **e** Calculated FS of the bulk states. **f** Photoelectron intensity plots of the band structure taken along the $\overline{\Gamma}$ - $\overline{K}$ - $\overline{M}$ direction on the Sn termination, measured with 74 *eV* linear horizontal (LH) (i) and linear vertical (LV) (ii) polarizations. **g** Same as (f), but measured with 88 *eV*. The momentum path is indicated by the red line in (d).

When cleaved along the (001) direction, ScV_6Sn_6 crystals exhibit four possible surface terminations, namely the V_3Sn (kagome), $ScSn^3{}_2$, Sn^2, and SnV_3 terminations [marked as kagome layer, ScSn and Sn layers in Fig. S1a(i)]. By using a small beam spot, we were able to resolve three sets of electronic structure in ScV_6Sn_6. Given the similarity in local environments of Sn atoms in the Sn^2 and SnV_3 terminations, especially when extra Sn forms on the Sn^2 and SnV_3 terminations, causing the Sn^2 and SnV_3 terminations to appear experimentally mixed and indistinguishable, we assign the three sets of electronic structure to the vanadium kagome, $ScSn^3{}_2$, and Sn terminations. The measured Fermi

surface (FS) on the cleaved sample surface is summarized in Figs. S1b-d. As previously established in GdV_6Sn_6 compound [1], we assign the FS shown in Fig. S1c to the ScSn layer based on the detected Sn 4*d* core level (Fig. 2H in the main text) and the observed topological Dirac surface states (TDSSs) (Figs. 2i, 3a, 3b, and Fig. S1c). The FS presented in Fig. S1b (also Fig. 2c) agrees well with the calculated FS contributed by V-3*d* states (Fig. S1e), leading us to suggest it belongs to the kagome layer. The FS shown in Fig. S1d is attributed to the Sn layer [Fig. S1a(ii)]. The band structure along the $\overline{\Gamma}$ - $\overline{K}$ direction collected on the Sn layer is shown in Figs. S1f and S1g.

Supplementary Note 2. K_z dispersion in ScV_6Sn_6

Density functional theory (DFT) calculations reveal a noticeable dispersion along the *c*-direction in ScV_6Sn_6 (Fig. S2a), in contrast to the relatively weak k_z dispersion in CsV_3Sb_5 [2]. To determine the k_z, we performed photon energy–dependent ARPES measurements on the kagome termination over a broad energy range (40 to 120 *eV*) (Fig. S2b). The experimental k_z (Fig. S2c) is determined by examining the dispersion of the band bottom of the electron band around *M* point in the out-of-plane momentum direction, as shown by the red curve in Fig. S2c(i) and the blue curve in Fig. S2c(ii).

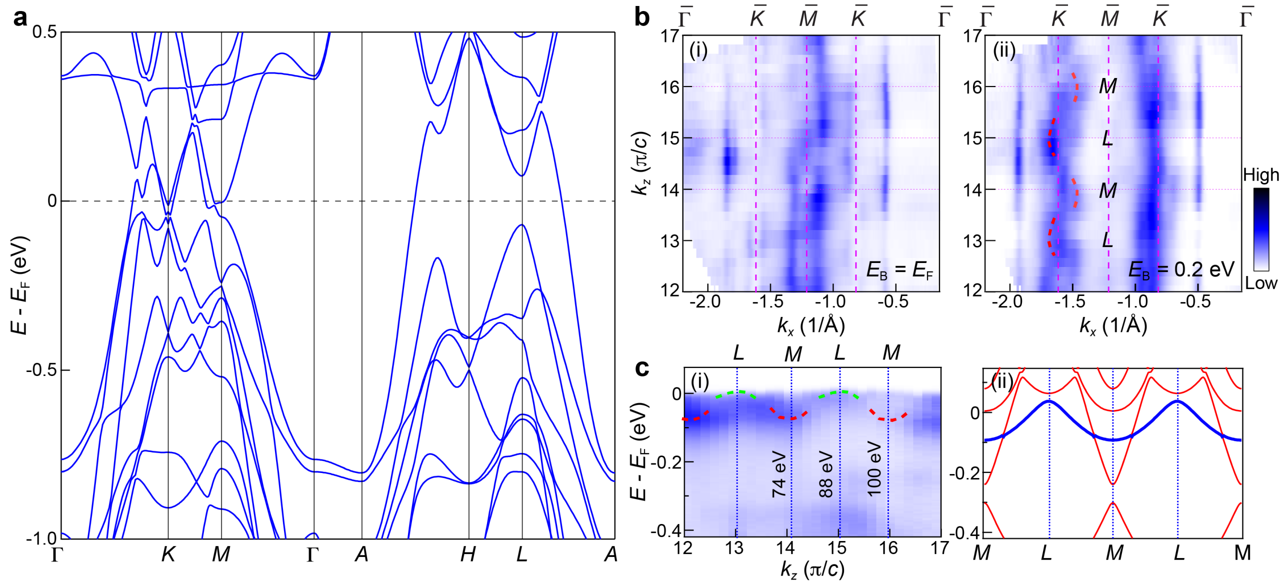

Fig. S2| K_z dispersion in ScV_6Sn_6. a DFT band structure of the pristine ScV_6Sn_6. **b** Photon energy-dependent ARPES spectral intensity map at the Fermi level (E_F) (i) and a binding energy (E_B) of 0.2 *eV* (ii), measured along the $\bar{\Gamma}$ - $\bar{K}$ direction. **c** The measured (i) and calculated (ii) band dispersions along the *M* - *L* direction.

Supplementary Note 3. $\mathbb{Z}_2$ topological surfaces states in ScV_6Sn_6

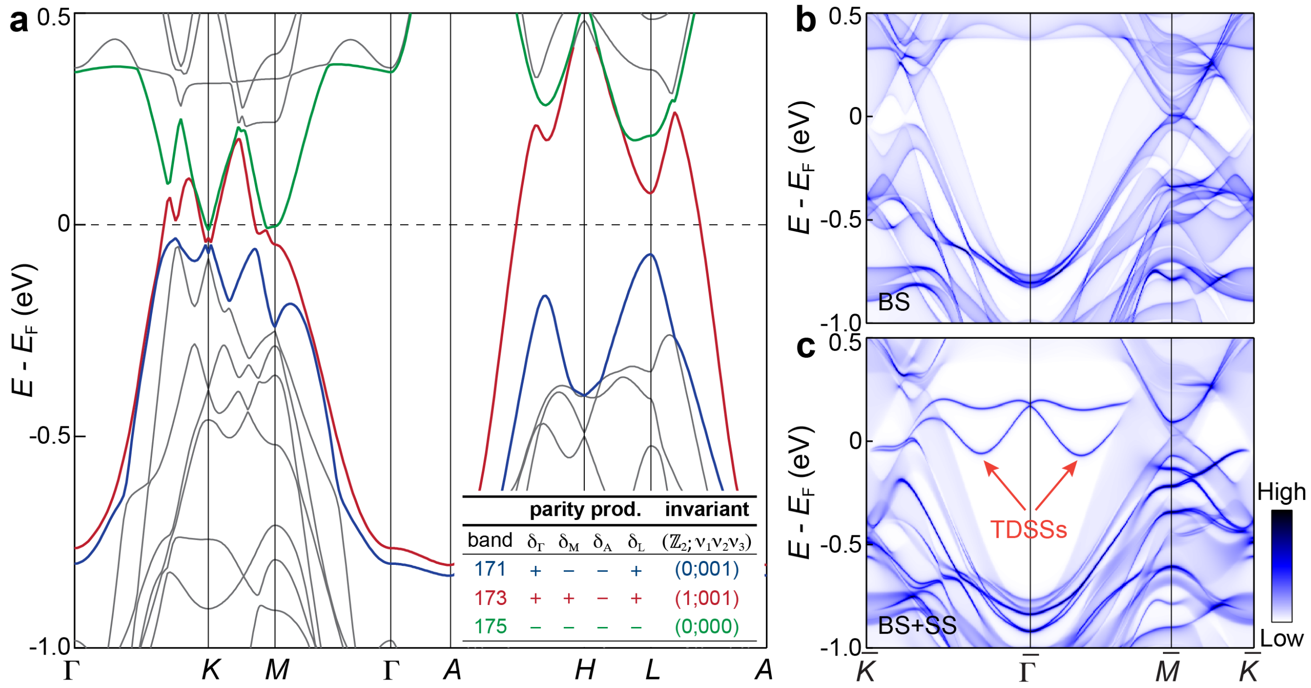

Fig. S3| $\mathbb{Z}_2$ topological surfaces states in ScV_6Sn_6. a DFT calculated electronic structure of the pristine ScV_6Sn_6. The inset shows the parity products and the $\mathbb{Z}_2$ invariant of different bands. Band 173 (red) is characterized by a strong topological invariant, $\mathbb{Z}_2$ =1, while band 171 (blue) and 175 (green) are trivial with no topological invariants. TDSSs are expected to reside in the large local band gap at Γ. **b, c** The (001) surface Green's function projection of pure bulk states (BS)(b) and the states comprising both BS and surface states (SS) on the ScSn termination (c). The red arrows highlight the TDSSs.

Supplementary Note 4. VHS around the *K* point

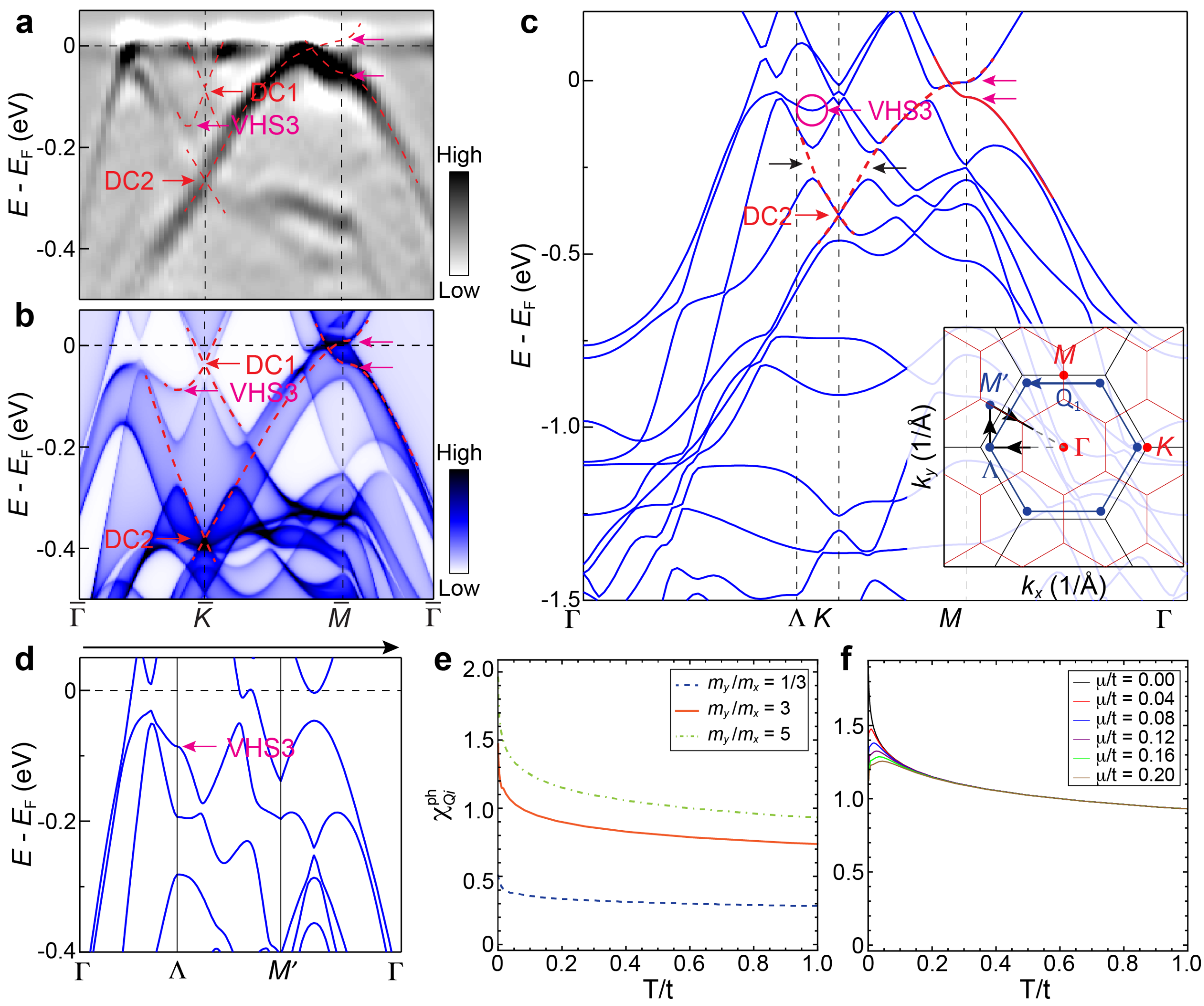

Fig. S4| Van Hove singularity around the *K* point. a Experimental band dispersion measured along the $\bar{\Gamma}$ - $\bar{K}$ - $\bar{M}$ - $\bar{\Gamma}$ direction. **b** Calculated band structure along the $\bar{\Gamma}$ - $\bar{K}$ - $\bar{M}$ - $\bar{\Gamma}$ direction, with red and pink arrows indicating the Dirac cone (DC) and Van Hove singularities (VHSs), respectively. **c** DFT calculated electronic structure along the Γ - K - M - Γ direction of the pristine ScV_6Sn_6. Schematics of the Brillouin zone and VHSs around the *K* point are indicated in the inset of (c). Black and red hexagons represent original BZ and CDW BZ, respectively. **d** DFT bands along the Γ - Λ - M' direction. The momentum path is indicated by the black arrow in (d). **e** Temperature-dependent susceptibilities in the particle-hole channel with the momentum transfer Q_1 for the VHSs around *K* point [indicated in (d)] for different effective mass ratios. **f** Same data as in (a), but for different chemical potentials with m_y/m_x=5.

The experimental band dispersion along the $\bar{\Gamma}$ - $\bar{K}$ - $\bar{M}$ - $\bar{\Gamma}$ direction (Fig. S4a and Fig. 3d in the main text) is overall good consistent with the calculations (Fig. S4b and Fig. 3e). However, it should be noted that while the DFT calculations show a hybridization gap on the Dirac cone (DC) band around the *K* point (indicated by the black arrow in Fig. S4c), this gap is absent in the ARPES spectrum (Fig. 4a). To further investigate the VHS nature around the Λ point, we plot the band dispersion along the Γ - Λ - M' - Γ direction (Fig. S4c), which clearly reveals a VHS (VHS3) around the Λ point, as shown in Fig. 4d. Figure 4e displays the particle-hole susceptibility at Q_1 as a function of temperature for different parameters (different mass ratios) when the VHS is located at the Fermi level (E_F). The

susceptibility increases rapidly with decreasing temperature, suggesting that nesting could induce a CDW instability at a low temperature. As VHSs move away from E_F, the corresponding charge fluctuation weakens but remains noteworthy (temperature-dependent susceptibilities for different chemical potential, as depicted in Fig. S4f). Although these charge fluctuations, enhanced by interactions, may not directly lead to a CDW, they can provide a secondary contribution to the phonon-induced CDW in ScV_6Sn_6.

Supplementary Note 5. Orbital character resolved band structure of ScV_6Sn_6

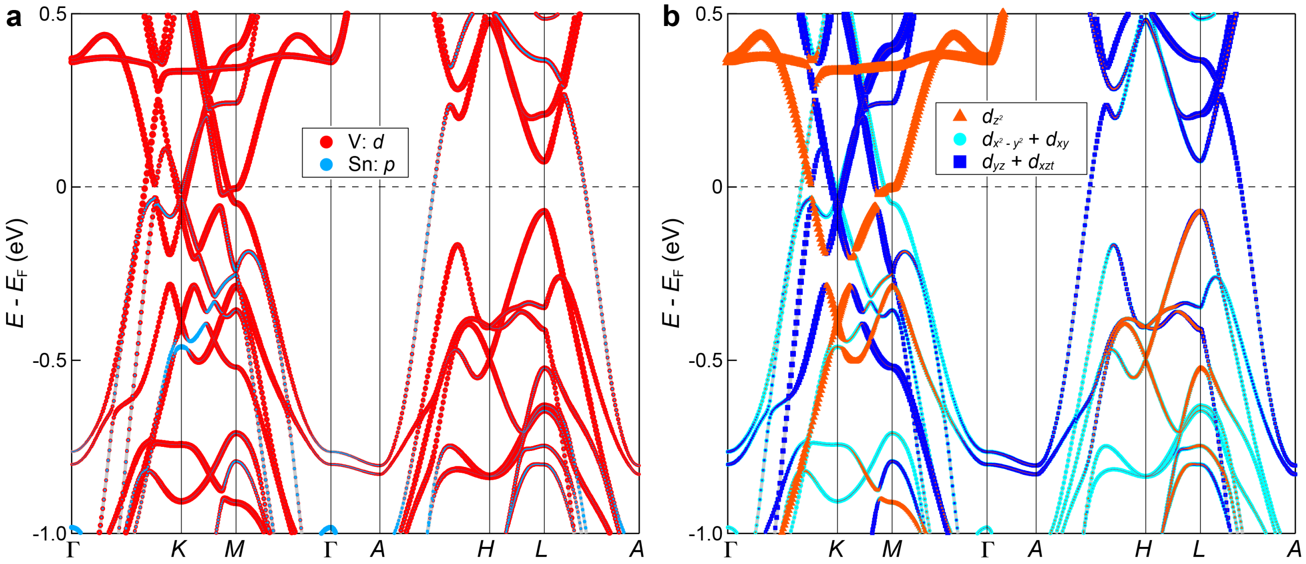

Fig. S5| Orbital character resolved band structure of ScV_6Sn_6. a Orbital projected band structure for the V d and Sn *p* orbitals. **b** Orbital projected band structure for the d orbitals of V atoms.

Supplementary Note 6. Evolution of the Raman modes in ScV_6Sn_6 across the CDW transition

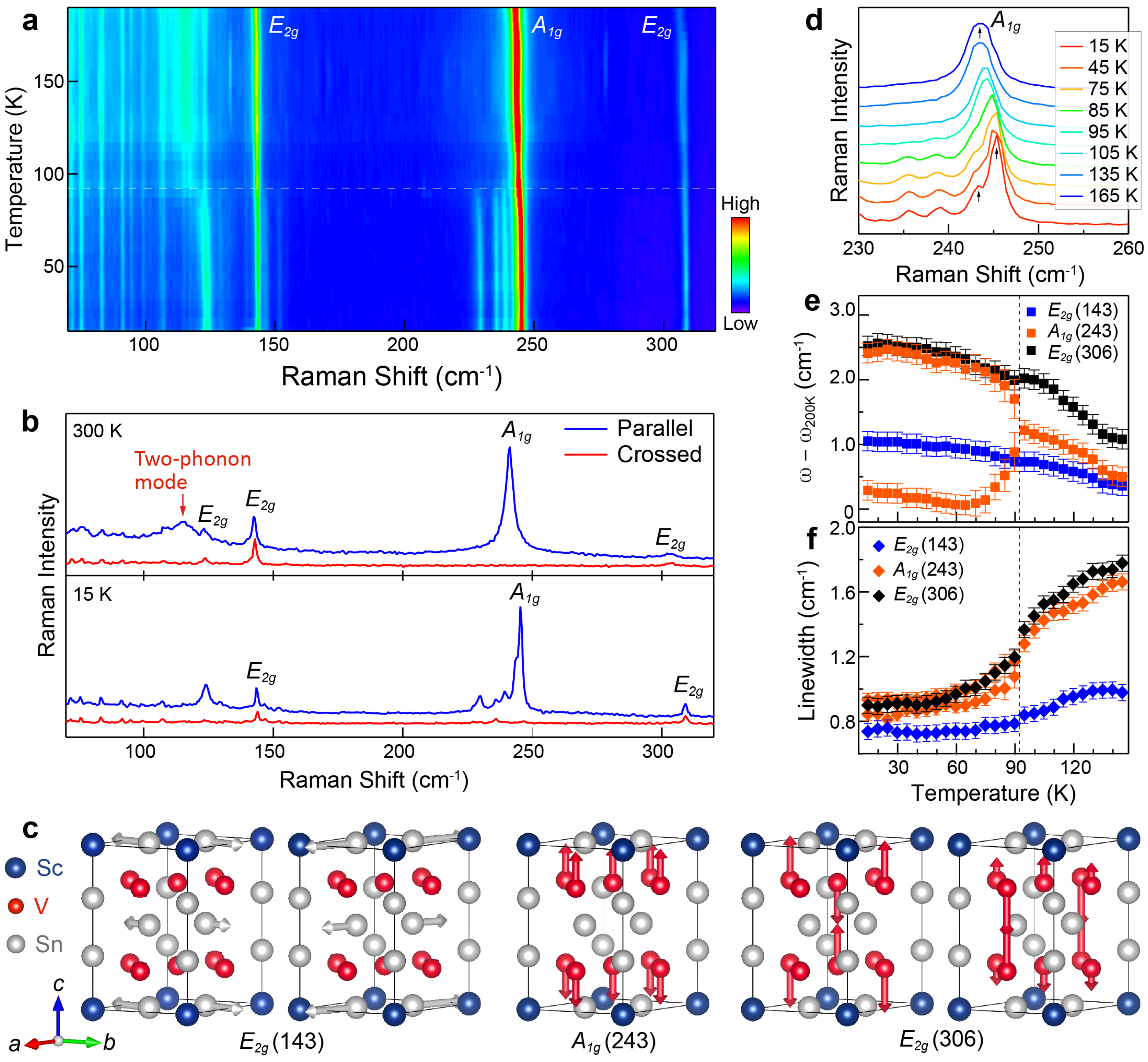

Fig. S6| Evolution of the Raman modes in ScV_6Sn_6 across the CDW transition. a Colormap of the Raman response recorded on ScV_6Sn_6 showing a temperature dependence, measured with parallel polarization. **b** Raman spectra measured on the *ab*-plane in parallel and crossed polarizations. **c** Crystal structure of ScV_6Sn_6, with different Wyckoff positions labeled. The vectors illustrate the vibration patterns of the main lattice E_{2g} (doubly degenerate) and A_{1g} modes. **d** Temperature-dependent Raman spectra illustrating the A_{1g} mode. The black arrows indicate the Raman peaks. **e**,**f** Evolution of frequency (e) and linewidth (f) for the E_{2g} and A_{1g} main lattice phonons. The frequency is compared to the corresponding values at 200 *K*.

Figure S6a presents a wide-range colormap of the Raman response, covering a Raman shift from 70 cm^{-1} to 320 cm^{-1}. Similar to CsV_3Sb_5 [3], multiple weak peak-like structures below 100 cm^{-1} are observed. However, these weak peaks exhibit almost no temperature dependence (Fig. S6a), likely originating from rotational spectrum of O_2 and N_2 along the laser path [4]. Figure S6b compares the Raman spectra measured on the *ab*-plane in parallel and crossed polarizations. According to the polarization selection rules in backscattered geometry, E_{2g} modes are active in both crossed and

parallel polarizations, and A_{1g} modes are only active in parallel polarization, while E_{1g} modes are not active in either polarization (only a very weak signal is observed in crossed polarization). Therefore, we attribute the two prominent Raman-active phonon peaks at 143 cm^{-1} and 243 cm^{-1}, mentioned in the main text, to the E_{2g} and A_{1g} modes, respectively. The former is attributed to the in-plane vibrations of Sn^3 atoms, and the latter is due to the out-of-plane vibrations of V atoms (Fig. S6c). Similarly, the phonon mode around 306 cm^{-1} is also assigned to E_{2g} (Fig. S6a and S6c).

Upon closer examination of the temperature-dependent Raman spectra, a double-peak feature of the A_{1g} mode in the CDW state is revealed (Fig. S6d). To understand its origin, we extract the frequency (with the corresponding value at 200 *K* subtracted, Fig. S6e) and linewidth (full width at half maximum, Fig. S6f) from Lorentzian fits of the peaks for the A_{1g} mode, as depicted in Figs. S6d and S6e. Remarkably, around the CDW phase transition temperature, the sum of the linewidth of the two single peaks below T_{CDW} is well above the single peak linewidth above T_{CDW}, suggesting a splitting of the A_{1g} mode in the CDW state. This finding indicates that CDW induces a modulation of the lattice along the *c*-axis [5], highlighting the crucial role of electron-lattice coupling in promoting the three-dimensional CDW [5]. Moreover, the extracted frequency (Fig. S6e) and linewidth (Fig. S6f) for the two E_{2g} modes show a kink feature, signifying that the E_{2g} modes are also influenced by the CDW transition. These results strongly imply an intimate link between the phonon dynamics and CDW promotion in ScV_6Sn_6.

We next discuss the potential scenarios could explain the appearance of the new emergent modes at low temperatures (Fig. S6a, and Fig. 4d in the main text).

1) **Modification of lattice symmetry:** This could allow for new modes or the splitting of existing ones. However, there is no evidence of other phase transitions reported by other techniques. Moreover, if it were a structural phase transition, additional modes would likely emerge from existing ones, which is not observed here.

2) **Zone folding:** This often results from an overlapping symmetry in the crystal lattice, such as charge density waves (CDW) or long-range magnetic order. In our case, it is likely that some of these modes arise from zone folding, given their proximity to optical phonon branches and their narrow width.

3) **Modes associated with lattice modulation itself:** These are new modes linked to the CDW. They can be of amplitude origin (equivalent to transverse optical modes) or in phase (equivalent to acoustic phonons). Both types are, in principle, visible due to the reduction of the Brillouin zone.

We have assigned new modes, such as the A_3 mode, as amplitude modes because they exhibit a soft mode behavior, a strong characteristic of amplitude collective excitations. While zone folding may play a role, these new modes in ScV_6Sn_6 behave more like amplitude modes with a very long coherence length. Depending on crystal quality and CDW stability, these amplitude modes may have very similar coherence to normal phonons and thus very similar widths. Moreover, these new modes with relatively weak softening in ScV_6Sn_6 (Fig. 4d in the main text) closely resemble the amplitude

modes suggested in CsV_3Sb_5 [3], where the peak shift is similarly minimal. The absence of complete softening to zero frequency could be related to the first-order CDW transition in these kagome metals. Nevertheless, achieving unambiguous identification would require further modeling, a scope beyond the present paper. Importantly, concerning the conclusions presented in our paper, distinguishing between an amplitude mode and zone folding is not consequential, as both scenarios showcase robust electron-lattice coupling.

Supplementary References:

[1] Hu, Y. *et al.* Tunable topological Dirac surface states and van Hove singularities in kagome metal GdV_6Sn_6. *Sci. Adv.* **8**, add2024 (2022).

[2] Hu, Y. *et al.* Rich nature of van Hove singularities in kagome superconductor CsV_3Sb_5. *Nat. Commun*. **13**, 2220 (2022).

[3] Liu, G. *et al.* Observation of anomalous amplitude modes in the kagome metal CsV_3Sb_5. *Nat. Commun*. **13**, 3461 (2022).

[4] Ohno, H., Iizuka, Y. & Fujita, S. Pure rotational Raman spectroscopy applied to N_2/O_2 analysis of air bubbles in polar firn. *J. of Glaciol.* **67**(265), 903–908 (2021).

[5] Hill, H. M. *et al.* Phonon origin and lattice evolution in charge density wave states. *Phys. Rev. B* **99**, 174110 (2019).
